# Protein Biomarkers for the Early Detection of Breast Cancer

**DOI:** 10.1155/2011/343582

**Published:** 2011-08-11

**Authors:** David E. Misek, Evelyn H. Kim

**Affiliations:** ^1^Department of Surgery, University of Michigan Medical School, Ann Arbor, MI 48109-5656, USA; ^2^The Comprehensive Cancer Center, University of Michigan Medical School, Ann Arbor, MI 48109-5656, USA

## Abstract

Advances in breast cancer control will be greatly aided by early detection so as to diagnose and treat breast cancer in its preinvasive state prior to metastasis. For breast cancer, the second leading cause of cancer-related death among women in the United States, early detection does allow for increased treatment options, including surgical resection, with a corresponding better patient response. Unfortunately, however, many patients' tumors are diagnosed following metastasis, thus making it more difficult to successfully treat the malignancy. There are, at present, no existing validated plasma/serum biomarkers for breast cancer. Only a few biomarkers (such as HER-2/neu, estrogen receptor, and progesterone receptor) have utility for diagnosis and prognosis. Thus, there is a great need for new biomarkers for breast cancer. This paper will focus on the identification of new serum protein biomarkers with utility for the early detection of breast cancer.

## 1. Introduction

Advances in breast cancer control will be greatly aided by early detection, thereby facilitating diagnosis and treatment of breast cancer in its preinvasive state prior to metastasis. Breast cancer is the most frequently occurring malignancy and the second leading cause of cancer-related death for women in the United States [1]. The most efficacious screening modality utilized in the clinic is mammography though lesions less than 0.5 cm in size remain undetectable by present technology. Importantly, however, even though a breast lesion may be detected, given the low sensitivity/specificity of mammography, approximately 4-fold more women (than those with breast malignancies) have resultant biopsies. Five-year survival of women with breast cancer is highly correlated with tumor stage, with tumor detection at very early stages (stages 0 and I) having an approximate 98% 5-year survival. Five-year survival for stage II tumors is approximately 85%, stage III approximately 60%, and stage IV approximately 20%. Overall, breast cancer has an approximate 80% 5-year survival, with 207,090 new cases and 39,840 deaths expected in women in the United States in 2010 [1]. 

Early detection of breast cancer does allow for increased treatment options, including surgical resection, with a corresponding better patient response. Surgical resection may involve lumpectomy or mastectomy with removal of some of the axillary lymph nodes. Following early detection, radiation therapy, chemotherapy (before or after surgery), and hormone therapy (tamoxifen [2] and aromatase inhibitors [3–5]) also have utility for therapeutic intervention. Targeted biologic therapy with trastuzumab (Herceptin) [6] or lapatinib (Tykerb) [7, 8] also has utility to treat HER2/neu-positive breast tumors. Unfortunately, however, in the absence of good serum/plasma biomarkers many breast cancer patients are diagnosed too late in the disease process (i.e., after the tumors metastasize) for surgical resection to be an effective option. Thus, these patients are typically offered various therapeutic treatment modalities dependent upon tumor subtype (ER^+^ or ER^−^; HER2^+^ or HER2^−^). The available treatment modalities may include hormonal (antiestrogen), taxane (docetaxel or paclitaxel) or nontaxane chemotherapy. In general, women with metastatic breast cancer are provided one therapeutic modality until treatment failure and are then switched to another therapeutic modality. 

The origin of most breast cancer cases is not known. However, many risk factors have been identified, including female gender, increasing patient age, family history of breast cancer at an early age, early menarche, late menopause, older maternal age at first live childbirth, prolonged hormone replacement therapy, exposure to therapeutic chest irradiation, benign proliferative breast disease, and genetic mutations in genes such as BRCA1/2 [9]. The overwhelming majority of breast masses detected by palpation and/or by mammography are epithelial lesions, which include benign fibrocystic change, hyperplasia, carcinoma in situ, and infiltrating mammary carcinoma. Although several histologic types and subtypes of mammary carcinomas exist, >95% are either ductal or lobular carcinomas [10], with the majority (75%–80%) of mammary carcinomas being ductal carcinomas [11, 12]. 

A number of genetic alterations have been identified in breast tumors. The most frequent genomic aberrations identified are gains along chromosomes 1q, 8q, 17q, 20q, and 11q and losses along 8p, 13q, 16q, 18q, and 11q [13–18]. Interestingly, many of these chromosomal segments harbor known proto-oncogenes and/or tumor suppressor genes such as BRCA1, BRCA2, HER2-neu, C-MYC, and Cyclin D-1. Low-grade (grade 1) infiltrating ductal carcinomas have relatively few numbers of chromosomal alterations with the highest frequency of aberrations occurring as losses on 16q and gains on 1q. 

It is generally accepted that estrogen receptor-positive (ER^+^) and ER-negative (ER^−^) breast cancers are two different disease entities. ER^−^ tumors tend to be of high grade, have more frequent p53 mutations, and have worse prognosis compared with ER^+^ disease. Both ER^+^ and ER^−^ tumors can be either HER2 positive or negative. Low-grade tumors are typically ER positive, almost always HER2 nonamplified, and frequently overexpress cyclin D-1 [10]. In contrast, high-grade (grade 3) tumors tend to be ER negative, have frequent loss of p53 function, usually overexpress C-MYC and commonly overexpress HER2 [13–15, 19]. In the high-grade tumors, loss of p53 function is usually due to 17p13 deletion, mutation or inactivation, while overexpression of HER2 is usually because of 17q12 amplification [20–28].

Although early detection of cancer has improved survival for a number of cancers, including breast cancer [29], colon cancer [30–32], prostate cancer [33, 34], and cervical cancer [35], existing serum biomarkers for breast cancer are not adequate for early detection. The possibility of early detection of breast cancer may be realized through both noninvasive (i.e., imaging technologies) and invasive means (patient serum profiling). To date, gains in the early detection of breast cancer have been largely made due to routine mammography and/or by palpation (either self-examination or by physician or nurse practitioner). Imaging technologies (mammography, digital mammography, and magnetic resonance imaging (MRI)) have been adopted clinically for mass screening purposes, but there is resistance for seeking such services on a yearly basis, given the relative complexity and high cost-to-benefit ratio of these imaging methodologies. As a result, there has been much interest in development and validation of serum-based biomarkers for the early detection, risk stratification, prediction, and disease prognosis of breast cancer. This paper will focus on recent developments in identification of new serum protein biomarkers with potential utility for the early detection of breast cancer (Table 1).

## 2. Autoantibodies and Breast Cancer

The humoral immune response to cancer in humans has been well demonstrated by identification of autoantibodies to a number of different intracellular and surface antigens in patients with various tumor types [36–39]. A tumor-specific humoral immune response directed against oncoproteins [40, 41], mutated proteins such as p53 [42, 43], or other aberrantly expressed proteins have all been described. While it is currently unknown whether the occurrence of such antibodies is beneficial, knowledge of potential tumor antigens that may evoke tumor-specific immune responses may have utility in early cancer diagnosis, in establishing prognosis and in immunotherapy against the disease. 

Several approaches are currently available for the identification of tumor antigens. In contrast to identification of tumor antigens based on analysis of recombinant proteins (which do not contain posttranslational modifications as found in tumors or tumor cell lines), it may be preferable to utilize a proteomics-based approach for the identification of tumor antigens. This may facilitate the identification of autoantibodies to naturally occurring proteins, such as in lysates prepared from tumors and tumor cell lines, and may uncover antigenicity associated with aberrant posttranslational modification of tumor cell proteins. Such a proteomics approach was implemented for the identification of breast tumor antigens that elicit a humoral response against proteins that are expressed in the SUM-44 breast cancer cell line. 2D PAGE was used to simultaneously separate individual cellular proteins from the SUM-44 cell line. The separated proteins were transferred onto PVDF membranes. Sera from breast cancer patients were screened individually for antibodies that reacted against the separated proteins by Western blot analysis. Proteins specifically reacting with sera from the breast cancer patients were identified by mass spectrometry. Le Naour and colleagues [36] have shown that a humoral response directed against RS/DJ-1 occurred in 13.3% of newly diagnosed breast cancer patients. None of the 25 healthy controls (0%) or 46 patients (0%) with hepatocellular carcinoma exhibited autoantibodies to RS/DJ-1. Only 2/54 (3.7%) samples of sera from lung adenocarcinoma patients demonstrated autoantibodies to RS/DJ-1. 

In breast cancer, besides RS/DJ-1 [36], autoimmunity has also been shown against a number of other cellular proteins. These proteins include p53 [44–47], heat shock protein 60 [48, 49], heat shock protein 90 [50, 51], and mucin-related antigens [49, 52–54]. The presence of p53 autoantibodies have been observed in 15% of patients with breast cancer and were shown to be associated with a poor prognosis [44, 45, 47]. However, p53 autoantibodies have also been found in patients with other malignancies and inflammatory conditions [42, 43], thus the humoral response to p53 is not specific to breast cancer. A humoral response to the 90 kDa heat shock protein has also been associated with poor survival in breast cancer [51]. In contrast, the presence of MUC1 autoantibodies has been associated with a reduced risk for disease progression in patients with breast cancer [53, 54]. While the antigenic epitope on MUC1 (or, for that matter, any of the other breast tumor antigens discussed above) is unknown, MUC1 has been shown to be aberrantly glycosylated frequently in breast cancer [54]. At present, CA 15-3 (a soluble or secreted form of MUC1) has utility as a circulating marker for breast cancer [55, 56]. Serial measurements of CA 15-3 have utility to detect recurrences and to monitor the treatment of metastatic breast cancer [55–57]. Additionally, the CA 15-3 concentration at initial presentation does have prognostic significance [58–62].

In order to circumvent many of the difficulties associated with 2D-PAGE (namely, inadequate resolution, slow throughput, and limited dynamic range), protein microarrays were developed that have the capability to screen patient's sera for autoantibodies directed against tumor antigens [63–66]. In comparison to traditional ELISAs that use single purified recombinant proteins, the protein microarrays are capable of presenting and analyzing >1000 tumor antigens simultaneously. In addition, as these tumor antigens are typically derived from diseased tissues or disease-related cells, they possess disease-related, potentially antigenic, posttranslational modifications not normally expressed by the particular cells or tissue. In this technology, proteins from diseased tissues or disease-related cell lines are separated by 2-dimensional liquid chromatography (chromatofocusing or ion exchange HPLC in the first dimension, followed by reverse phase HPLC in the second dimension). Following separation, all fractions (≥1700 fractions) from each separation are printed onto nitrocellulose-coated microscope slides and are subsequently probed with sera from patients or control subjects [63–66]. As each reactive fraction may contain a number of different proteins, each reactive fraction would need to be further assessed to determine the tumor antigen of interest.

More recently, Ramachandran et al. [67, 68] developed a novel protein microarray technology, termed nucleic acid protein programmable array (NAPPA). NAPPA arrays are generated by printing full-length cDNA encoding the target proteins at each feature of the array. The proteins are then transcribed and translated by a cell-free system and immobilized *in situ* using epitope tags fused to the proteins. Although this technology circumvents many of the difficulties of traditional protein microarrays (i.e., the need to resolve complex protein lysates), the printed proteins on the array lack all normal posttranslational modifications. Thus, any antigenicity resulting from aberrant modification of tumor proteins is not assessed. Anderson and colleagues [69] utilized the NAPPA arrays to screen 4988 candidate tumor antigens with sera from patients with early stage breast cancer for autoantibodies. Twenty-eight of these antigens were confirmed using an independent serum cohort (*n* = 51 cases/38 controls, *P* < 0.05). Using all 28 antigens, a classifier was identified with a sensitivity of 80.8% and a specificity of 61.6% (AUC = 0.756). Although the sensitivity and specificity are not high, these 28 recombinant protein antigens may be considered as potential biomarkers for the early detection of breast cancer. 

It is not clear why only a subset of patients with a particular tumor type develop a humoral response to particular tumor antigens. Immunogenicity may depend on the level of expression, posttranslational modification, or other types of protein processing, the extent of which may be variable among tumors of a similar histological type. Other factors that may influence the immune response include variability among tumors and individuals in major histocompatibility complex molecules and in antigen presentation. Although a number of autoantibodies have been identified in breast cancer, in most cases, they occur in less than 50% of patient's sera. Therefore, they are not likely to be effective individually for the early detection of breast cancer but may show efficacy if utilized as a panel of biomarkers.

## 3. Detection of Altered Plasma Protein Expression for Identification of Breast Cancer-Specific Biomarkers

There has been great interest in the hypothesis that tumor-specific proteins may be found in patient's circulation, and they may have utility for the early detection of cancer. For example, proteins such as CA125 in ovarian cancer and prostate-specific antigen (PSA) in prostate cancer have been used clinically as diagnostic markers of cancer. CA125 is a mucin commonly employed as a diagnostic marker for epithelial ovarian cancer. PSA is secreted primarily by prostate epithelial cells into the seminal plasma and is one of the best characterized examples of a secreted glycoprotein used in cancer diagnostics. 

There are a number of reports that have described aberrantly expressed proteins in the serum of breast cancer patients. The most widely used serum marker in breast cancer diagnostics is CA 15-3, which detects soluble forms of the mucin MUC1. MUC1 is normally found in the apical membrane of normal secretory epithelium. Following malignant transformation, however, MUC1 may be localized throughout the external surface of the entire plasma membrane. In addition, changes in MUC1 glycosylation have been reported during neoplastic transformation [70, 71]. Although MUC1 is expressed in normal and neoplastic breast epithelium, the clinical utility of MUC1 measurements is confined to measurements of shed or soluble forms (termed CA 15-3), released from the cell surface by proteolytic cleavage. Unfortunately, CA 15-3 is not suitable for early detection, as serum levels are rarely increased in patients with early or localized breast cancer. The main utility for CA 15-3 is for monitoring therapy in patients with metastatic breast cancer.

Le Naour and coworkers [36] have evaluated RS/DJ-1 as a serum biomarker of breast cancer. In normal tissue, expression of RS/DJ-1 was observed in epithelium, smooth muscle, blood vessels, and nerves. All 15 (100%) invasive ductal carcinomas and 3 (100%) invasive lobular carcinomas showed some level of cytoplasmic and nuclear reactivity in the neoplastic cells. Significantly elevated levels of serum RS/DJ-1 was observed in the sera of 11/30 patients with newly diagnosed breast cancer, as compared to serum from 25 healthy subjects. However, these authors did not evaluate serum RS/DJ-1 levels in patients with other types of breast lesions. Thus, it is unknown whether the increased serum RS/DJ-1 levels are cancer-specific.

In another study [72], significantly higher serum HER-2/neu levels were found in patients with tissue overexpression of HER-2/neu. Univariate analysis showed that HER-2/neu serum levels were prognostic factors in disease-free survival and overall survival only in patients with tissue overexpression. When only patients with HER-2/neu overexpression in tissue were studied, tumor size, nodal involvement, and tumor markers (at least one positive) were found to be independent prognostic factors for both disease-free survival and overall survival.

## 4. Use of Mass Spectrometric Methodologies for Identification of Breast Cancer-Specific Biomarkers

Methodologies have been developed to directly analyze the proteins contained within complex protein mixtures, such as that found within human biofluids (plasma or serum, nipple aspirate fluid, ductal lavage fluid, saliva, etc.). Among these technologies, some, like SELDI (Surface-Enhanced Laser Desorption and Ionization) are mass spectrometry-based. A number of investigators have used SELDI-TOF mass spectrometry to interrogate serum [73–81] and nipple aspirate/ductal lavage fluid [82–89] from patients with breast cancer. In one study, serum samples from women with or without breast cancer were analyzed using SELDI protein chip mass spectrometry [77]. Using a case-control study design, serum samples from 48 female patients with primary invasive breast cancer were compared with samples from 48 age- and sex-matched healthy controls. To increase the number of identifiable proteins, patient's serum was profiled on IMAC30 (activated with nickel) ProteinChip surfaces. Differences in protein intensity between breast cancer cases and controls were measured by the Mann-Whitney *U* test and adjusted for confounding variables in a multivariate logistic regression model. Three peaks, with mass-to-charge ratio (*m/z*) 4276, 4292 and 8941 were found that showed significant decreased expression in cancer sera, as compared to control sera (*P *< 0.001). One drawback of the SELDI technology, however, is that given the limited dynamic range of SELDI, it is likely that distinctive features observed in serum with this approach represent relatively abundant proteins that are not necessarily specific to breast cancer. Further, SELDI has difficulties in providing the identification of the distinctive proteins when used to directly profile complex protein mixtures.

## 5. Mass-Spectrometric Profiling of Nipple Aspirate Fluid or Ductal Lavage Fluid

Other mass spectrometric profiling methods have been utilized to profile proteins found in nipple aspirate fluid [90] and ductal lavage fluid in order to identify breast cancer-specific biomarkers. These investigators [90] analyzed paired nipple aspirate fluid samples from 18 women with stage I or stage II unilateral invasive breast cancer and 4 healthy volunteers using ICAT (isotope-coded affinity tag) labeling, followed by SDS-PAGE. Gel slices were cut from each sample, with subsequent analysis by liquid chromatography tandem mass spectrometry (LC-MS/MS). They identified 353 peptides from the tandem mass spectra. Alpha-2-HS-glycoprotein was found to be underexpressed in nipple aspirate fluid from tumor-bearing breasts, while lipophilin B, beta-globin, hemopexin and vitamin D-binding protein were all overexpressed. Unfortunately, these authors only identified abundant proteins whose over- or underexpression was somewhat modest. Moreover, these authors did not analyze nipple aspirate fluid from patients with inflammatory breast disease. Thus, conclusions cannot be drawn regarding breast cancerspecificity of protein expression.

## 6. N-linked Glycan Profiling for Biomarker Identification in Breast Cancer Serum

Glycoproteins are the most heterogeneous group of posttranslational modifications known in proteins. Glycans show a high structural diversity reflecting inherent functional diversity. N- and O-oligosaccharide variants on glycoproteins (glycoforms) can lead to alterations in protein activity or function that may manifest itself as overt disease [91, 92]. Many clinical biomarkers and therapeutic targets in cancer are glycoproteins [93–95], such as CA125 in ovarian cancer, HER2/neu in breast cancer, and prostate-specific antigen (PSA) in prostate cancer. The human epidermal growth factor receptor 2 (HER2/neu) is a transmembrane glycoprotein, where the presence of HER2 overexpression appears to be a key factor in malignant transformation and is predictive of a poor prognosis in breast cancer. CA125 is a mucin commonly employed as a diagnostic marker for epithelial ovarian cancer. Although CA125 has been used as an ovarian cancer marker for a long time, many of its O- and N-glycan structures have only recently been characterized [96]. PSA is secreted primarily by prostate epithelial cells into the seminal plasma. It is one of the best characterized examples of a secreted glycoprotein used in cancer diagnostics, and its glycoforms have been described [97]. The alteration in protein glycosylation that occurs through varying the heterogeneity of glycosylation sites or changing glycan structure of proteins on the cell surface and in body fluids has been shown to correlate with the development or progression of cancer and other disease states [98]. It has been reported that the glycosylation of PSA secreted by the tumor prostate cell line LNCaP differs significantly from that of PSA from seminal plasma (normal control). These carbohydrate differences allow a distinction to be made between PSA from normal and tumor origins and provide a valuable biochemical tool for diagnosis of prostate cancer [99]. 

There is growing evidence that glycan structures on glycoproteins are modified in breast cancer [100–109]. Breast cancer-associated alterations have been demonstrated for fucosylation groups and for sialylations on the plasma protein *α*-1-proteinase inhibitor [106]. Increased GlcNAc *β*1-6Man *α*1-6Man *β*-branching in asparagine-linked oligosaccharides has been observed in human tumor cells. The levels of the *β*1-6 branched oligosaccharides were evaluated in a series of benign and malignant human breast biopsies. Normal human breast tissue and benign lesions showed low expression but 50% of the primary malignancies examined showed significantly elevated *β*1-6 branching [107]. Subsequently, L-PHA (a lectin that binds specifically to the *β*1-6 branched oligosaccharides) lectin histochemistry was performed on paraffin sections of human breast tissues. All breast carcinomas and epithelial hyperplasia with atypia demonstrated significantly increased L-PHA staining as compared to fibroadenomas and hyperplasia without atypia [108]. More recently, L-PHA reactive glycoproteins were identified from matched normal (nondiseased) and malignant tissue isolated from patients with invasive ductal breast carcinoma [109]. Comparison analysis of the data identified 34 proteins that were enriched by L-PHA fractionation in tumor relative to normal tissue for at least 2 cases of ductal invasive breast carcinoma. Of these 34 L-PHA tumor enriched proteins, 12 were common to all 4 matched cases analyzed.

Abd Hamid and coworkers [110] analyzed fluorescently tagged serum *N*-glycans of advanced breast cancer patients using exoglycosidases and LC-MS/MS. They found that the expression of a trisialylated triantennary glycan containing an *α*-1,3-linked fucose was increased in the presence of breast cancer. Kyselova and coworkers. profiled the permethylated *N*-glycans in sera of breast cancer patients at different stages (stages I to IV) using MALDI TOF/TOF MS in one study [111]. In a second study, they profiled reduced and methylated serum *N*-glycans of late-stage breast cancer patients using nanoliquid chromatography (LC) chip/time-of-flight (TOF) MS [112]. In both studies, they found an increase in fucosylation in both core and branched segments of *N*-glycans in the presence of breast cancer. In the latter study, they found a decrease in expression of a biantennary-monosialylated *N*-linked glycan and an increase in expression of a fucosylated triantennary-trisialylated *N*-linked glycan in the presence of Stage IV breast cancer. These glycosylation changes in a tumor-secreted protein may reflect fundamental activity changes in the enzymes involved in the glycosylation pathway, either through altered levels of enzymes or altered enzymatic activity. Importantly, the changes in glycan structure may serve as early detection biomarkers of breast cancer.

## 7. Summary

Early detection of breast cancer, so as to diagnose and treat cancer in its preinvasive state prior to metastasis, may greatly impact the treatment and prognosis of patients with this common, but deadly, malignancy. Unfortunately, at present, suitable biomarkers have not been identified for the early detection of breast cancer. Biomarker discovery for this disease is still very much in its discovery phase. Multiple approaches have been developed, as described above, that hold promise for the identification of serum biomarkers. The protein biomarkers that have been identified to date do not possess the requisite sensitivity/specificity to have utility individually as a biomarker for the early detection of breast cancer but ultimately may have utility within a panel of protein biomarkers. Additionally, other emerging technologies, such as genetically engineered mouse models of breast cancer may have utility to identify panels of serum biomarkers that can be further explored in human sera. In order to determine the utility of any promising protein biomarkers, the candidates will need to be tested and validated by multiple independent studies using an adequately sized test and training set of sera samples from very early-stage breast cancer. Development of such resources, including serum from patients with nonmalignant breast lesions and prospective serum collection from individuals at high risk of being diagnosed with breast cancer as well as serum from patients with other breast lesions and other types (nonbreast) of malignancies is of critical need for the identification of biomarkers with utility for the early detection of breast cancer. Up until now, serum/plasma collection has been primarily performed in individual laboratories, using heterogeneous sample collection methods. The Human Proteome Organization (HUPO) has conducted a study to assess efficacious serum collection methods. These findings have lead to efforts presently being made by the National Cancer Institute, through the Early Detection Research Network, to develop suitable serum resources for both the discovery phase and the subsequent validation phase of biomarkers for the early detection of cancer. With the ultimate development of these standardized resources, it is expected that suitable biomarkers would be validated and have utility for the early clinical detection of breast cancer within the next five-to-ten years.

## Figures and Tables

**Table 1:** Current promising biomarkers for the detection of breast cancer.

Name of biomarker	Technology used for discovery	Type	Reference
RS/DJ-1	Humoral response	autoantibody	[36]
p53	Humoral response	autoantibody	[44–47]
HSP60	Humoral response	autoantibody	[48, 49]
HSP90	Humoral response	autoantibody	[50, 51]
Mucin-related	Humoral response	autoantibody	[49, 52–54]
CA 15-3	Serum profiling	serum protein	[55, 56]
RS/DJ-1	Serum profiling	serum protein	[36]
HER-2/neu	Serum profiling	serum protein	[72]
*α*-2-HS-glycoprotein	Nipple aspirate fluid profiling	Ductal protein	[90]
Lipophilin B	Nipple aspirate fluid profiling	Ductal protein	[90]
beta-globin	Nipple aspirate fluid profiling	Ductal protein	[90]
Hemopexin	Nipple aspirate fluid profiling	Ductal protein	[90]
Vitamin D-binding protein	Nipple Aspirate Fluid Profiling	Ductal protein	[90]
